# Is the Direct Anterior Approach for Total Hip Arthroplasty Effective in Obese Patients? Early Clinical and Radiographic Results from a Retrospective Comparative Study

**DOI:** 10.3390/medicina59040769

**Published:** 2023-04-16

**Authors:** Alberto Di Martino, Niccolò Stefanini, Matteo Brunello, Barbara Bordini, Federico Pilla, Giuseppe Geraci, Claudio D’Agostino, Federico Ruta, Cesare Faldini

**Affiliations:** 1 I Orthopedic and Traumatology Clinic, IRCCS Istituto Ortopedico Rizzoli, 40136 Bologna, Italy; 2 Department of Biomedical and Neuromotor Science-DIBINEM, University of Bologna, 40136 Bologna, Italy; 3 Medical Technology Laboratory, IRCCS Istituto Ortopedico Rizzoli, 40136 Bologna, Italy

**Keywords:** Total hip arthroplasty, Direct Anterior Approach, obese, overweight, outcomes

## Abstract

*Background and objectives:* Total hip arthroplasty (THA) in obese patients (BMI > 30) is considered technically demanding, and it is associated with higher rates of general and specific complications including infections, component malpositioning, dislocation, and periprosthetic fractures. Classically, the Direct Anterior Approach (DAA) has been considered less suitable for performing THA surgery in the obese patient, but recent evidence produced by high-volume DAA THA surgeons suggests that DAA is suitable and effective in obese patients. At the authors’ institution, DAA is currently the preferred approach for primary and revision THA surgery, accounting for over 90% of hip surgeries without specific patient selection. Therefore, the aim of the current study is to evaluate any difference in early clinical outcomes, perioperative complications, and implant positioning after primary THAs performed via DAA in patients who were divided according to BMI. *Material and methods:* This study is a retrospective review of 293 THA implants in 277 patients that were performed via DAA from 1 January 2016 to 20 May 2020. Patients were further divided according to BMI: 96 patients were normal weight (NW), 115 were overweight (OW), and 82 were obese (OB). All the procedures were performed by three expert surgeons. The mean follow-up was 6 months. Patients’ data, American Society of Anesthesiologists (ASA) score, surgical time, days in rehab unit, pain at the second post-operative day recorded by using a Numerical Rating Scale (NRS), and number of blood transfusions were recorded from clinical charts and compared. Radiological evaluation of cup inclination and stem alignment was conducted on post-operative radiographs; intra- and post-operative complications at latest follow-up were recorded. *Results:* The average age at surgery of OB patients was significantly lower compared to NW and OW patients. The ASA score was significantly higher in OB patients compared to NW patients. Surgical time was slightly but significantly higher in OB patients (85 ± 21 min) compared to NW (79 ± 20 min, *p* = 0.05) and OW patients (79 ± 20 min, *p* = 0.029). Rehab unit discharge occurred significantly later for OB patients, averaging 8 ± 2 days compared to NW patients (7 ± 2 days, *p* = 0.012) and OW patients (7 ± 2 days; *p* = 0.032). No differences in the rate of early infections, number of blood transfusions, NRS pain at the second post-operative day, and day of post-operative stair climbing were found among the three groups. Acetabular cup inclination and stem alignment were similar among the three groups. The perioperative complication rate was 2.3%; that is, perioperative complication occurred in 7 out of 293 patients, with a significantly higher incidence of surgical revisions required in obese patients compared to the others. In fact, OB patients showed a higher revision rate (4.87%) compared to other groups, with 1.04% for NW and 0% for OW (*p* = 0.028, Chi-square test). Causes for revision in obese patients were aseptic loosening (2), dislocation (1), and clinically significant post-operative leg length discrepancy (1), with a revision rate of 4/82 (4.87%) during follow-up. *Conclusions:* THA performed via DAA in obese patients could be a solid choice of treatment, given the relatively low rate of complications and the satisfying clinical outcomes. However, surgical expertise on DAA and adequate instrumentation for this approach are required to optimise the outcomes.

## 1. Introduction

Obesity is defined as a Body Mass Index (BMI) higher than 30 kg/m^2^ [1]. This condition has exponentially increased in the last decade in developed countries, predisposing patients to multiple pathologies, including diabetes, dyslipidaemia, and heart attack [2,3,4]. Among orthopaedic conditions, obese people are at a high risk of developing osteoarthritis, which generally occurs at a younger age when compared to non-obese patients, due to the mechanical overload of the lower limbs caused by excessive weight [5,6]. In end-stage osteoarthritis, joint replacement surgery is associated with acceptable risks and a good chance of recovery of function.

Total hip arthroplasty (THA) is regularly carried out in obese patients, with more than one third of implants performed in this patient population [5,7]. However, it is well known that obesity-associated comorbidities may hamper the operability of patients and that THA in obese patients is associated with an overall increase in intra- and peri-operative complications; among these, the most severe include infections, component malpositioning leading to implant instability and dislocation, and periprosthetic femur fractures [8].

The optimal surgical approach to minimise intra- and post-operative complications in obese patients is still under debate [9,10]. Reconstructive orthopaedic surgeons all over the world generally favour posterolateral and direct lateral approaches, mainly because these are considered easier to extend to better expose the acetabulum and femur in case of intra-operative complications. The Direct Anterior Approach (DAA) is used in THA to reduce the invasiveness of surgery and to promote a faster post-operative recovery [11,12,13]. However, the use of DAA to perform THA in obese patients is greatly debated in the literature. Although according to several authors [14,15,16,17], DAA in the obese patient is contraindicated because of the worse outcomes when compared to other surgical approaches, more recent studies [18,19,20] performed in institutions with high-volume DAA THAs show that obese patients could significantly benefit from the reduced invasiveness of DAA in terms of blood loss, operating time, and functional recovery [19].

At the authors’ institution, DAA is currently the preferred surgical approach for primary and revision THA surgery, accounting for over 90% of the procedures, and it is performed without a specific restriction with respect to body weight. Therefore, the aim of the current study is to evaluate any potential difference in early clinical outcomes, perioperative complications, and implant positioning after primary THAs performed via DAA, in patients divided according to BMI.

## 2. Materials and Methods

**Figure 1:**
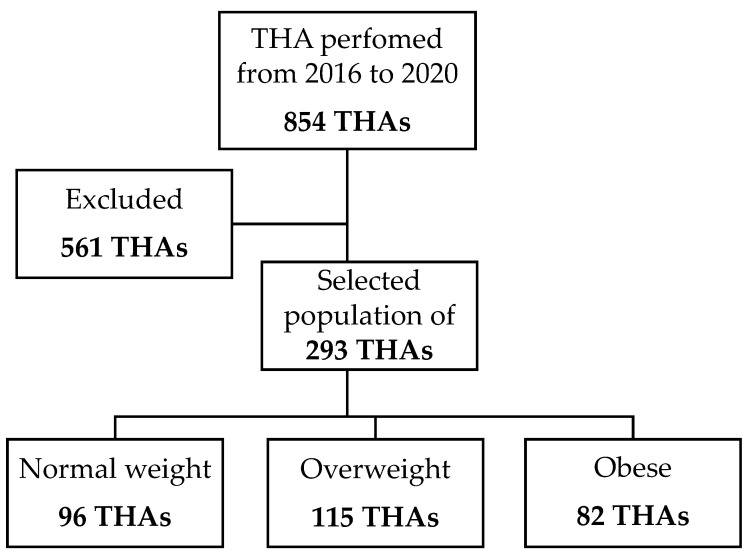
Flow-chart of the study.

The current study and all the case collections were approved by the Local Ethical Committee (CE-AVEC) with the code 021 ANT-HIP, 347/2021/Oss/IOR. Patients were considered for inclusion retrospectively if they were operated on for primary THA from 1 January 2016 to 20 May 2020 at the authors’ institution (Figure 1). Inclusion criteria were as follows: patients affected by primary osteoarthritis and treated with THA through DAA, and a follow-up of at least 6 months. Exclusion criteria were patients treated with approaches other than DAA and patients operated on for secondary osteoarthritis to avoid possible bias given by pre-existing clinical conditions including hip deformity, trauma, and infection that could hamper THA outcomes. All the procedures were performed by three expert senior surgeons of the unit. The average follow-up was 6 months. At the acetabular level, all the patients were implanted with a VERSAFIT-Cup or MPACT-Cup (Medacta International, Castel San Pietro, Switzerland); at the femur site, implants were AMIStem (Medacta International, Castel San Pietro, Switzerland).

Patients were subsequently divided into 3 groups according to their BMI: normal weight (BMI < 25 kg/m^2^, NW), overweight (25 kg/m^2^ ≤ BMI ≤ 30 kg/m^2^, OW), and obese (BMI > 30 kg/m^2^, OB). Demographic and clinical parameters were retrospectively collected from the medical records of the hospital: age at surgery, American Society of Anesthesiologists (ASA) score, surgical time (from surgical incision to end of suture), in-hospital length of stay, pain at the second post-operative day recorded by using a Numerical Rating Scale (NRS), day of post-operative stair climbing, and number of blood transfusions were recorded and compared. Any intra- and post-operative complications were recorded while the patients were in hospital and at outpatient evaluations performed at 1, 3, and 6 months post-operatively. 

### 2.1. Surgical Technique

**Figure 2:**
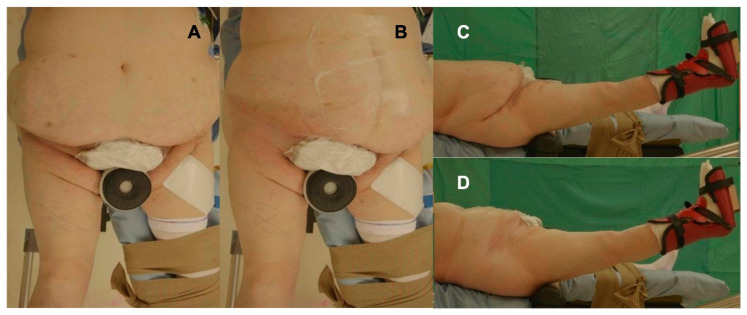
(**A**) Coronal view of patient positioning on the traction table to perform THA via DAA. (**B**) Coronal view, in which adipose tissue is displaced from the point of incision with an adhesive drape. (**C**) Sagittal view of patient positioning on the traction table; the leg is placed in a boot and is positioned with the leg at 30° of flexion, with neutral abduction and rotation. The belly fat is placed over the incision site. (**D**) Sagittal view, in which the incision site is free; adipose tissue is moved on contralateral side with adhesive drape.

The surgical technique for THA performance through DAA was the same for each patient independently of the body habitus. All patients received prophylactic antibiotic therapy with 2 g of intravenous cefazolin. Patients were positioned supine with the foot of the operated leg secured by a boot on a specific traction table managed by a non-scrubbed assistant, which allows for the control of traction, rotation, and adduction or abduction (Figure 1). The patient’s lower limb was positioned with the leg at 30° of flexion, with neutral abduction and rotation (Figure 2). In obese patients, the adipose tissue at the abdomen is generally placed anteriorly and above the inguinal ligament [21], as it represents an obstacle for the surgical incision; it is displaced towards the contralateral side, being anchored by using an adhesive drape to improve the exposure at the surgical site (Figure 2A,B).

**Figure 3:**
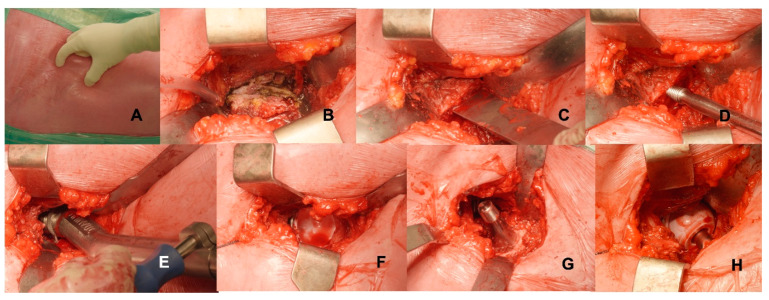
(**A**) DAA incision is performed 2 cm distally and 2 cm laterally from the anterior superior iliac spine; (**B**) through the intermuscular space between the tensor fascia latae and sartorious and rectus femoris muscles, the capsula is completely exposed. (**C**) Neck osteotomy is performed with the head in situ; it is performed with an oscillating saw, and it is completed via an osteotome, which allows mobilisation of the head of the femur. (**D**) The femoral head is removed with a corkscrew, with the leg in increased traction and external rotation. (**E**) Reaming of the acetabulum is performed with increasing reamers; in the anterior approach, the use of off-setted handles and instrument is required to improve accuracy and to decrease the impingement against the soft tissues. (**F**) The exposure of the acetabular cup via the anterior approach is optimal and allows a full view of the cup and ceramic liner. (**G**) Femur is exposed in full extension, external rotation, and adduction; after femoral broaching, the definitive stem implant is inserted at the femoral shaft. (**H**) Ceramic head is impacted, and joint reduction is performed via limb abduction, flexion, and internal rotation.

The surgical incision was performed 2 cm distally and 2 cm laterally from the anterior superior iliac spine averaging 7–9 cm (Figure 3). After subcutaneous tissue dissection, fascia was cut over the belly of the tensor fascia lata muscle to minimise the risk of injury to the lateral femoral cutaneous nerve. Subsequently, the intermuscular and interneural space was dissected by blunt dislocation of the muscles: the tensor fascia lata muscle was retracted laterally, and the sartorius and the rectus femoris were retracted medially. Branches of the lateral circumflex artery can be ligated or coagulated. The capsula was completely exposed and carefully opened, creating a thick flap reflected proximally, and it was sutured at the end of surgery. Osteotomy of the femoral neck was performed with the head in situ by using an oscillating saw. The head was then removed by using a corkscrew, with the leg in slight traction and external rotation to widen the space of the osteotomy. Acetabular and femoral bones were prepared for implant positioning by using dedicated instrumentation; handles of the reamer and of the broaches should be curved and off-set to ease bone preparation and to avoid impingement against the soft tissues. Iliofemoral and pubofemoral ligaments were incised to improve proximal femur exposure. Accurate posterior–medial capsular release at the proximal femur was performed before femoral broaching, which requires the patient’s leg to be positioned in external rotation, extension, and adduction. After the positioning of cup and femur implants, a reduction manoeuvre was performed, and a suture was started.

### 2.2. Post-Operative Care

After surgery, pain medications were administered according to hospital protocols to promote a faster recovery. Weight-bearing is allowed at patients’ tolerance with the aid of crutches. At the authors’ institution, patients remained in-hospital after surgery to begin rehabilitation, which was assisted by the physical therapists. It started on the same day of the surgery, or the day after if THA was performed in the afternoon. Patients were encouraged to sit as soon as the epidural analgesia wore off, and they were helped to stand upright as soon as they felt comfortable to do so. Isometric exercises with active knee extension were promoted, together with flexion–extension of the ankle, both to be independently performed in bed. The patient was educated on the prevention of movements that could promote dislocation, namely, adduction combined with hyperextension and external rotation. Stair climbing was allowed as soon as the patient gained walking autonomy, and it was set as a goal from the second day after surgery. As soon as the patient was clinically stable and able to walk autonomously with crutches, to climb stairs with aids, to get dressed, and to access the bathroom independently, they were discharged home or were moved to another facility.

### 2.3. Radiological Evaluation

**Figure 4:**
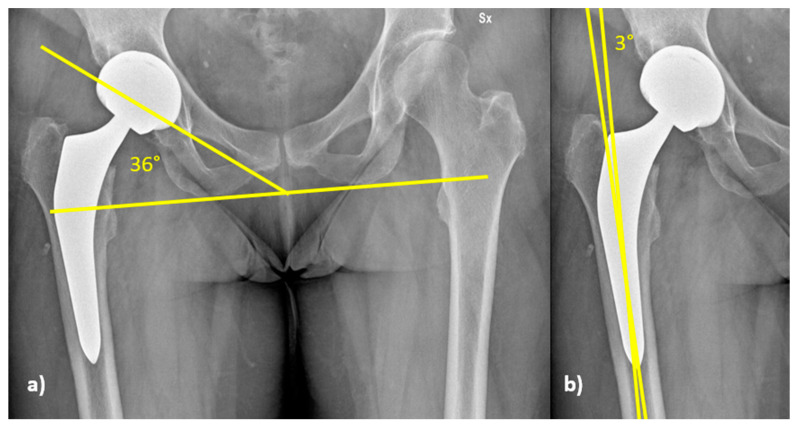
(**a**) Cup inclination is measured as the angle between the line tangent to the border of the acetabular cup and a line parallel to the horizontal plane. (**b**) Stem alignment is the angle between the axis of the stem and of the femur.

Radiological evaluation was performed on standard post-operative radiographs to evaluate cup inclination and stem alignment (Figure 4). Cup inclination (Figure 4a) was compared to Lewinnek’s safe zone, which is 40° ± 10° [22]. Stem alignment (Figure 4b) was considered good when the angle between the axis of the stem and that of the femur was 0° ± 5°; above or below the range, the implant alignment was considered in varus or valgus, respectively [23].

### 2.4. Statistical Analysis

Study variables were analysed and compared among groups. Comparison among the groups was performed by using Kruskal–Wallis and Chi-square tests (SPSS 14.0, version 14.0.1; SPSS Inc, Chicago, IL, USA). Significance was set at *p*-value < 0.05.

## 3. Results

From a total of 549 primary THAs performed from 1 January 2016 to 20 May 2020, 293 implants in 277 patients (145 males and 132 females; 52% M and 48% F) matched the inclusion criteria and were retrieved for analysis; according to BMI, 96 out of 293 implants were allocated in the NW group (41 males and 55 females; 43% M and 57% F), 115 were allocated in the OW group (69 males and 46 females; 60% M and 40% F), and 82 were allocated in the OB group (56% males and 44% females).

### 3.1. Patients’ Demographics and Characteristics

Age at surgery was significantly different between groups (*p* = 0.005, Kruskal–Wallis test) (Table 1). In particular, OB patients were significantly younger at surgery, averaging 58.6 years (range 36–83) when compared to OW patients (63.9 yo; range 34–86; *p* = 0.006, Kruskal–Wallis test) and NW patients (63.3 yo; range 34–86; *p* = 0.028, Kruskal–Wallis test). OB patients had significantly worse ASA scores at surgery compared to NW and OW patients (*p* = 0.009, Chi-square test). Sex distribution in different groups did not show significative differences.

Surgical time was slightly but significantly higher in OB patients (86 ± 21 min) compared to NW (79 ± 20 min, *p* = 0.05) and OW patients (80 ± 20 min) (*p* = 0.029; Kruskal–Wallis test). The overall incidence of perioperative complications was 2.3%, occurring in 7 out of 293 patients (Table 2). No difference in the overall rate of surgery-related complications was found among groups (*p* = 0.1868, Chi-square test), with an incidence of 4.87%, compared to 2.08% in NW and 0.87% in OW patients; the incidence of infections did not show difference in complication rate (*p* = 0.27540, Chi-square test). The two intra-operative femur fractures occurred in one NW and one OW patient, and these were intra-operatively managed by wiring cerclages. OB patients showed a higher revision rate (4.87%) compared to other groups, with 1.04% for NW and 0% for OW (*p* = 0.028, Chi-square test). Two OB patients experienced recurrent early dislocations in the first month after surgery; in both cases, the first episode occurred after a fall. One patient was managed via stem revision to correct anteversion, and the other via cup repositioning to correct inclination and anteversion of the implant. Two patients showed aseptic loosening of the femur, which was managed via stem revision. One OB patient experienced early wound dehiscence, which was managed via surgical revision and targeted antibiotic therapy (Table 2).

The time of in-hospital stay of the patients was significantly longer for OB patients, averaging 8 ± 2.4 days, compared to 7 ± 1.8 days for NW patients (*p* = 0.021, Kruskal–Wallis test) and 7 ± 2.2 days for OW patients (*p* = 0.032, Kruskal–Wallis test).

No differences in the number of blood transfusions were observed when the three groups were compared (*p*= 0.28), with NW patients requiring transfusions in 22% (23/96), OW requiring transfusions in 15% (13/115), and OB patients requiring transfusions in 22% (26/82). No differences in the rate of early infections were found among the three groups (*p* = 0.27, Chi-square test). No other significant differences were found when comparing the three groups in terms of pain at the second post-operative day recorded by using Numerical Rating Scale (NRS) and day of post-operative stair climbing (Table 3).

### 3.2. Radiographic Analysis

The analysis on cup inclination showed no significant differences among groups; in particular, cup inclination in the three groups had the same average value (34°), with standard deviations of 6.2° in NW, 7.2° in OW and 7.9° in OB patients (*p* = 0.571, Kruskal–Wallis, test). Acetabular cup inclination was within the safe zone in 222 patients (75.8%): 74/96 (77.1%) in NW group, 86/115 (74.8%) in the OW group, and 62/82 (75.8%) in OB patients. No significant differences were found comparing the distributions of acetabular cup inclination according to Lewinnek (*p* = 0.927, Chi-square test); 71/293 (24.2%) patients had acetabular cup inclination outside the safe zone, being below in 67/71, and above in 4/71; 3 out of these 4 patients were in the OB group (Table 4).

The three groups showed the median value for stem alignment of 0°, with standard deviations of 2.2° in NW, 2.2° in OW, and 2.0° in OB. In the NW group, all the stems (96 patients) were positioned in the range of tolerance. In the OW group, 113/115 stems were positioned in the range, and 2/115 stems were above the tolerance value, with 1 being in valgus of 9.4° and 1 being in varus of 5.4°. In OB patients, 80/82 stems were implanted in the range. In total, 2 out of 82 stems in the OB patients were beyond the range, both being aligned in valgus of 5.4° and 7°, respectively. No significant difference in the distribution among the groups was observed for stem alignment (*p* = 0.943, Chi-square test).

## 4. Discussion

Our study retrospectively evaluated the early outcomes and complications of THAs performed through DAA in 277 consecutive patients divided into 3 groups according to their BMI: normal weight (NW), overweight (OW), and obese (OB). OB patients had a lower age at surgery and higher ASA score, together with a slightly but significantly longer surgical time and in-hospital stay. A significantly higher number of revision surgeries was observed in patients in the OB group, which were due to dislocation (n = 2), wound dehiscence (n = 1), and aseptic mobilisation (n = 1) (*p* = 0.028); interestingly, no significant differences (*p* = 0.27) in the incidence of infections was found when comparing obese and non-obese patients.

Patients in the OB group were significantly younger compared to NW and OW patients (59.47 vs. 64.02 and 63.59, respectively). These data are in accordance with the observations of Haynes [24] and Clement and Deehan [25], who found that patients with a BMI > 30 kg/m^2^ were subjected to primary THA in their early sixties compared to non-obese patients that were closer to their seventies, supporting the role of obesity in the development of early hip arthritis. OB patients in our study also showed higher ASA scores compared to non-obese patients, in agreement with data from the literature [26].

Surgical time for THA implant in OB patients was significantly longer (86 ± 21 min) compared to NW (79 ± 20 min) and OW (80 ± 20 min) patients; this finding is in line with others available in the literature [27,28,29], in which surgical time for DAA in obese patients is, on average, 10.9 min above the average time for non-obese patients (104.0 vs. 115.9 min). However, the slight increase in surgical time in our OB patients, which is less than 10 min compared to NW and OW, has a low impact from a clinical point of view, as mirrored by the absence of difference in the rate of post-operative infections and blood transfusion requirements among the three groups. Sang et al. [28] reported a significant increase in blood loss in obese patients undergoing THA, often requiring allogeneic blood transfusions; DAA in the OB patients might be of advantage in this scenario since, according to our findings, no difference in blood transfusions between obese and non-obese patients was found in patients operated on via this minimally invasive approach.

In our study, the in-hospital stay was slightly but significantly longer in OB patients, in disagreement with the findings by Hartford et al. [29], who reported similar results in NW and OB patients (2.7 vs. 2.81 days). In the current study, however, patients were not discharged until they gained functional autonomy; the average 1-day-longer hospital stay for OB patients could be related to the increase in the overall comorbidities in this patient population, and not just to the surgery itself.

Several authors reported a higher rate of surgery-related complications in obese patients operated on for THA. DeMik et al. [8] investigated a pool of 64,648 patients that had THA, 37.48% of whom were obese. They found that OB patients having THA had higher rates of complications compared to the non-obese, including wound complications (1.53% vs. 0.72%), deep infections (0.58% vs. 0.24%), and reoperations (2.11% vs. 1.59%). When patients are operated on through DAA, intra-operative femoral fractures and implant malpositioning could occur because of the impingement of the surgical instruments against the adipose tissue [14,16,30]. In order to reduce periprosthetic fracture, specific short stems seem to reduce the risk; in addition, dedicated off-set instrumentation is required to permit a suitable placement of implants without force [31]. Russo et al. [14] reported an increased rate of infectious complications in patients with BMI over 30 kg/m^2^. In our study, we found a higher overall number of complications requiring revision surgery but no statistically significant differences in the rate of infections comparing the 3 groups: only 1 out of 72 patients had a wound infection requiring revision surgery, an incidence lower than previously reported [14]. In addition, Avinash et al. reported a low rate of infection, specifically, of 0.58%, in a study of more than 800 THAs in a population with an average BMI of 28 [32]. These data could support the protective role of minimally invasive DAA against infections, even in high-risk OB patients [33]. However, considering the restricted number of patients of the examined cohort, there is the risk of underestimating the rate of complications. We could not find any difference in terms of blood loss, implant positioning, and intra- and post-operative complications; our data are in agreement with the findings of Argyrou et al. [19], who, in a population of 82 OB patients compared to 172 NW patients undergoing THA surgery, did not find significant differences in blood loss, intra- and post-operative complications, or implant positioning and alignment. Intra-operative femur fracture is traditionally reported as the main complication of DAA in OB patients, with a reported incidence of up to 8.4% of cases [34,35,36]; however, in our patient population, no perioperative femur fractures occurred in the OB group.

The radiological evaluation of implant positioning in the current study focused on cup inclination and stem alignment. Cup inclination was analysed with respect to Lewinnek’s safe zone of 40° ± 10° [22]. The relevance of Lewinnek’s safe zone as an outcome measurement of THA has been recently discussed [37]. The results of the current study seem to support this issue: in fact, even with a non-negligible number of patients (see Table 2) with cup inclination outside the “safe zone”, a low incidence (2/293, 0.68%) of implant dislocations was found. Measured cup inclination was comparable among the three groups, showing that DAA allows a consistent implant positioning, independently of the body habitus. This is in line with the findings of Davidovitch et al. [38], who, in a study on 509 fluoroscopy-assisted DAA THAs divided into 3 groups according to BMI, Group I (<30 kg/m^2^), Group II (≥30 to <35 kg/m^2^), and Group III (≥35 kg/m^2^), did not find significant differences in acetabular component positioning when obese and non-obese patients were compared. Good stem positioning (range −5°/+5°) was observed in most patients, with a prevalence of mild varus stems (167/293; 57%); the data are in agreement with the findings of Haversath et al. [39], who reported the same prevalence of mild varus (+2.2°) stem alignment when THA was performed via DAA.

This study has some limitations. The first is associated with its retrospective nature, which is associated with a selection bias. Moreover, collected patients were operated on in a surgical unit with great expertise on DAA, which is currently used in over 90% of THAs for both primary and revision surgeries. The follow-up was short and focused only on early results, reaching up to 6 months; however, since the purpose of the study was to evaluate the feasibility and safety of DAA in OB compared to non-obese patients, follow-up was no longer deemed necessary to support the study questions. Moreover, surgery-related complications, including dislocations, occur in over 50% of cases in the first 3 months after surgery [40].

## 5. Conclusions

In conclusion, the results of the current study support the performance of minimally invasive DAA for THA in obese patients by surgeons experienced in this approach, because it can improve the outcomes of THA surgery in this patient population [27,41,42]. Thanks to its low invasiveness, THA performed via DAA is associated with good early functional outcomes and with an acceptable rate of complications. For those reasons, this approach should be taken into account by the hip surgeon when treating an obese patient.

## Figures and Tables

**Table 1:** Surgery-related parameters.

Patients Characteristics	NW (n = 96)	OW (n = 115)	OB (n = 82)
Age (Years)	63.3 (34–86 years)	63.9 (34–86 years)	58.6 (36–83 years)
Sex (M/F)	43% M/57% F (41 M 55 F)	60% M/40% F (69 M 46 F)	56% M/44% F (46 M 36 F)
ASA I	39% (37)	23% (26)	16% (13)
ASA II	47% (45)	62% (71)	63% (52)
ASA III	14% (14)	15% (18)	21% (17)

**Table 2:** Complications that occurred in the study group.

Complications	NW	OW	OB	Total
Wound dehiscence	-	-	1/82 (1.22%)	1/293 (0.34%)
Intra-operative femur fracture	1/96 (1.04%)	1/115 (0.87%)	-	2/293 (0.68%)
Dislocation	-	-	2/82 (2.43%)	2/293 (0.68%)
Aseptic loosening	1/96 (1.04%)	-	1/82 (1.22%)	2/293 (0.68%)
Total	2/96 (2.08%)	1/115 (0.87%)	4/82 (4.87%)	7/293 (2.3%)

**Table 3:** Secondary clinical parameters.

	NW	OW	OB	
NRS at 2nd post-operative day	1.2 ± 1.0	1.3 ± 1.1	1.5 ± 1.0	*p* = 0.346
Day of post-operative stair climbing	3.4 ± 1.7	3.5 ± 1.9	3.7 ± 1.7	*p* = 0.456

**Table 4:** Distribution of acetabular cup inclination.

	NW	OW	OB	Total
Within Lewinnek’s safe zone	74 (77.1%)	86 (74.8%)	62 (75.6%)	222 (75.8%)
Out of Lewinnek’s safe zone	22 (22.9%)	29 (25.2%)	20 (24.4%)	71 (24.2%)
Total	96	115	82	293

## Data Availability

Not applicable.

## Abbreviation

THA	Total hip arthroplasty
DAA	Direct Anterior Approach
BMI	Body Mass Index
NW	Normal weight patients
OW	Overweight patients
OB	Obese patients
ASA	American Society of Anesthesiologists
NRS	Numerical Rating Scale
